# Supplementary material for: Dietary Zinc and Risk of Prostate Cancer in Spain: MCC-Spain Study
Source: Nutrients. 2018 Dec 20;11(1):18. doi: 10.3390/nu11010018 (PMC6356690; doi:10.3390/nu11010018)
Supplement: Supplementary file 1 [file nutrients-11-00018-s001.zip › Supplementary table S1.pdf]

Table S1: Odds Ratio of prostate cancer a) adjusted by age, education, BMI, family history of prostate cancer, calcium intake, grains and legumes consumption, energy intake, physical activity (meets/day), smoking and alcohol intake as fixed effects and province of residence as a random effect, and b) also adjusted by having ever had taken supplements with zinc.

			ALL[a] n=1938			ALL[b] n=1834
**ZINC**	**Controls**	**Cases**	**OR[c](95%CI)**	**Controls**	**Cases**	**OR[d](95%CI)**
**T1**	404	215	1.00	364	214	1.00
**T2**	404	245	1.17 (0.89;1.54)	371	244	1.13 (0.86;1.50)
**T3**	399	271	1.38 (0.97;1.97)	370	271	1.34 (0.94;1.92)
**p-trend**			0.073			0.108
