# Supplementary material for: Dietary Zinc and Risk of Prostate Cancer in Spain: MCC-Spain Study
Source: Nutrients. 2018 Dec 20;11(1):18. doi: 10.3390/nu11010018 (PMC6356690; doi:10.3390/nu11010018)
Supplement: Supplementary file 1 [file nutrients-11-00018-s001.zip › Supplementary table S2.pdf]

Table S2. Association between dietary zinc and prostate cancer according to ISUP and AJCC classifications

			International Society of Urological Pathology grading (ISUP)					American Joint Committee on Cancer (8th Edition Classification)				
			1-2 n=546		3-5 n=174			I-IIA n=321		IIB-IV n=384		
	**Co**	**Ca**	**RRR(95%CI)**	**Ca**	**RRR(95%CI)**	**p-het**	**Ca**	**RRR(95%CI)**	**Ca**	**RRR(95%CI)**	**p-het**
**ZINC**						0.567					0.480
**T1** ***(<8.34mg/d)***	409	159	1.00	54	1.00		85	1.00	129	1.00	
**T2** ***(8.34-10.53 mg/d)***	409	178	1.18 (0.88;1.58)	62	1.21 (0.78;1.89)		111	1.35 (0.94;1.93)	124	1.06 (0.76;1.46)	
**T3** ***(>10.53 mg/d)***	410	209	1.50 (1.04;2.16)	58	1.20 (0.69;2.11)		128	1.65 (1.06;2.56)	133	1.26 (0.84;1.89)	
**p-trend**			0.028		0.523			0.026		0.264	

Relative risk ratio of prostate cancer adjusted by age, education, BMI, family history of prostate cancer, calcium intake and grains and legumes consumption as fixed effects and province of residence as a random effect.
