# Supplementary material for: Dietary Zinc and Risk of Prostate Cancer in Spain: MCC-Spain Study
Source: Nutrients. 2018 Dec 20;11(1):18. doi: 10.3390/nu11010018 (PMC6356690; doi:10.3390/nu11010018)
Supplement: Supplementary file 1 [file nutrients-11-00018-s001.zip › Supplementary table S3.pdf]

Table S3. Association between dietary zinc and prostate cancer by tumour aggressiveness and extension, adjusting by self-reported PSA screening

			Gleason=6 n=330		Gleason>6 n=386			cT1-cT2a n=574		cT2b-T4 n=108	
	**Co**	**Ca**	**RRR(95%CI)**	**Ca**	**RRR(95%CI)**	**p-het**	**Ca**	**RRR(95%CI)**	**Ca**	**RRR(95%CI)**	**p-het**
**ZINC**						0.278					0.777
**T1** ***(<8.34mg/d)***	359	83	1.00	129	1.00		170	1.00	36	1.00	
**T2** ***(8.34-10.53 mg/d)***	366	114	1.30 (0.89;1.89)	124	0.95 (0.67;1.34)		193	1.10 (0.81;1.50)	33	0.89 (0.51;1.57)	
**T3** ***(>10.53 mg/d)***	365	133	1.62 (1.02;2.57)	133	1.11 (0.72;1.72)		211	1.31 (0.88;1.93)	39	1.11 (0.56;2.22)	
**p-trend**			0.043		0.631			0.182		0.771	

Relative risk ratio of prostate cancer adjusted by age, education, BMI, family history of prostate cancer, calcium intake, grains and legumes consumption and psa as fixed effects and province of residence as a random effect.
