# Supplementary material for: Dietary Zinc and Risk of Prostate Cancer in Spain: MCC-Spain Study
Source: Nutrients. 2018 Dec 20;11(1):18. doi: 10.3390/nu11010018 (PMC6356690; doi:10.3390/nu11010018)
Supplement: Supplementary file 1 [file nutrients-11-00018-s001.zip › Supplementary table S4.pdf]

Table S4. Correlation between food groups and dietary zinc intake in MCC-Spain.

**Food groups**	
High-fat dairy	0.23
Low fat dairy	0.31
Eggs	0.24
White meat	0.29
Red meat	0.57
Processed meat	0.39
White fish	0.14
Oily fish	0.25
Seafood/shellfish	0.34
Leafy vegetables	0.25
Fruiting vegetables	0.33
Root vegetables	0.22
Other vegetables	0.36
Legumes	0.15
Potatoes	0.24
Fruits	0.29
Nuts	0.29
Refined grains	0.26
Whole grains	0.08
Olives and vegetable oil	0.38
Other edible fats	0.28
Sweets	0.23
Sugary	0.16
Juices	0.13
Caloric drinks	0.10
Convenience food and sauces	0.30
